# Repositioning the boundaries between public and private healthcare providers in the English NHS

**DOI:** 10.1108/JHOM-12-2018-0355

**Published:** 2019-11-07

**Authors:** Rod Sheaff, Joyce Halliday, Mark Exworthy, Alex Gibson, Pauline W. Allen, Jonathan Clark, Sheena Asthana, Russell Mannion

**Affiliations:** 1 School of Criminology, Law and Government, University of Plymouth, Plymouth, UK; 2 Health Services Management Centre, Birmingham University, Birmingham, UK; 3 HSRU, London School of Hygiene and Tropical Medicine, London, UK

**Keywords:** National Health Service, Public sector reform, Privatization, Ownership, Corporations, Not-for-profit organizations

## Abstract

**Purpose:**

Neo-liberal “reform” has in many countries shifted services across the boundary between the public and private sector. This policy re-opens the question of what structural and managerial differences, if any, differences of ownership make to healthcare providers. The purpose of this paper is to examine the connections between ownership, organisational structure and managerial regime within an elaboration of Donabedian’s reasoning about organisational structures. Using new data from England, it considers: how do the internal managerial regimes of differently owned healthcare providers differ, or not? In what respects did any such differences arise from differences in ownership or for other reasons?

**Design/methodology/approach:**

An observational systematic qualitative comparison of differently owned providers was the strongest feasible research design. The authors systematically compared a maximum variety (by ownership) sample of community health services; out-of-hours primary care; and hospital planned orthopaedics and ophthalmology providers (*n*=12 cases). The framework of comparison was the ownership theory mentioned above.

**Findings:**

The connection between ownership (on the one hand) and organisation structures and managerial regimes (on the other) differed at different organisational levels. Top-level governance structures diverged by organisational ownership and objectives among the case-study organisations. All the case-study organisations irrespective of ownership had hierarchical, bureaucratic structures and managerial regimes for coordinating everyday service production, but to differing extents. In doctor-owned organisations, the doctors’, but not other occupations’, work was controlled and coordinated in a more-or-less democratic, self-governing ways.

**Research limitations/implications:**

This study was empirically limited to just one sector in one country, although within that sector the case-study organisations were typical of their kinds. It focussed on formal structures, omitting to varying extents other technologies of power and the differences in care processes and patient experiences within differently owned organisations.

**Practical implications:**

Type of ownership does appear, overall, to make a difference to at least some important aspects of an organisation’s governance structures and managerial regime. For the broader field of health organisational research, these findings highlight the importance of the owners’ agency in explaining organisational change. The findings also call into question the practice of copying managerial techniques (and “fads”) across the public–private boundary.

**Originality/value:**

Ownership does make important differences to healthcare providers’ top-level governance structures and accountabilities and to work coordination activity, but with different patterns at different organisational levels. These findings have implications for understanding the legitimacy, governance and accountability of healthcare organisations, the distribution and use power within them, and system-wide policy interventions, for instance to improve care coordination and for the correspondingly required foci of healthcare organisational research.

## Repositioning the public–private boundary: supposed managerial consequences

### Neo-liberal “reform” repositions the public–private boundary

Since the 1980s, neo-liberal “reform” has in many countries shifted services across the boundary between the public and private sector. In the UK that boundary was successively brought closer to the core clinical activity of the English National Health Service (NHS) (see Box I).

The few NHS services denationalised outright were community health services (CHS), placed on the public–private boundary as “social enterprises” (managerially independent non-profit providers whose assets remained state owned but which could still go bankrupt) rather than as for-profit corporations. More often, NHS services’ staff, assets, activity and contract were in effect transferred across the public–private boundary to a non-NHS organisation (rather than expect the latter to assemble a new workforce and physical

Box I **Main stages of repositioning the public–private boundary in the NHS**

Privatised hospital ancillary services (cleaning, catering, laundry) (1984).

Creation of an ‘internal market’ (1991).

Waiting List Initiatives (1990s) introduced a minority of non-NHS providers of hospital services.

The Primary Care Act opened up primary medical care to commercial providers (1998).

The Private Finance Initiative: turn-key projects for non-NHS firms to design and build NHS hospitals, and operate their support services (1994).

Independent Sector Treatment Centres – corporate providers under NHS contract (2004).

Extending the internal market to community health services, which transferred from being Primary Care Trust managed to contractors (2004).

“Any Willing Provider” policy gave non-NHS bodies the right to bid to supply NHS-funded clinical care (2012).

Commissioning Support Units (consultancy-like support for NHS payers) (2012).

Alongside these changes, NHS service providers were reconstituted as “Foundation Trusts” with more business-like structures and, ostensibly, more autonomy from central government control.

infrastructure from scratch). National regulations protected NHS pay and conditions for the transferred staff. Policy statements announcing these changes (Department of Health, 2010) repeatedly presented them as (so to speak) “back-end” changes with no adverse implications for patients because NHS-funded care remained largely free at the point of use. At least for the purposes of persuading patients and voters, the public–private boundary was no longer defined in terms of provider ownership but financially, in terms of the source of payment for NHS services and, insofar as all providers operated under a common regulatory, monitoring and accountability frameworks (Department of Health, 2010), in terms of governance (Perry and Rainey, 1988).

### Ownership and organisation

This policy re-opens an old question about what differences, if any, arise between healthcare providers under different ownership. What do these differences imply for the legitimacy, governance and accountability of provider organisations, for the distribution and use of power within them and for system-wide policy interventions, for instance to improve care coordination?

This paper considers the connections between ownership, organisational structure and managerial regime within a larger theory linking those factors with the organisational environment, work processes and policy-relevant outputs (Sheaff *et al.*, 2004). It elaborates Donabedian’s (1980) reasoning about organisational structures to include organisations’ ownership, defined as the *de facto* control of that organisation’s activity, plant and products: that is, in terms of property, which “is in fact achieved in many different ways” (Bencherki and Bourgoin 2017), not only through legal or contractual rights. This implies that an organisation’s structure embodies, enacts and reproduces that ownership. Through the organisation’s structure, its owners, or their agents, select, implement and operate work processes (e.g. healthcare processes) chosen to realise the owners’ goals, and successively modify them through innovations intended to meet their goals more fully. When differently owned organisations select different production technologies one would predict the emergence of correspondingly differently constituted work groups and differently behaving work groups, insofar as work groups are characterised by their tasks and organisational context (Hackman, 1990). Structures and managerial practices for work coordination would correspondingly differ. Depending on the owners’ managerial skill, the organisation’s environmental circumstances and perhaps even an element of luck, the work processes would then produce outcomes which approximated to the owners’ original goals. For short, we label this conceptualisation as being part of a yet-to-be-developed ownership theory of organisations. In some ways akin to property rights theories of the firm (Kim and Mahoney, 2005), it is, however, intended as an empirical, explanatory theory outside the normative “social welfare” frameworks of neo-classical economics and, therefore, of transaction cost or the structure–conduct–performance theory (McWilliams and Smart, 1993).

It is also a multi-level analysis (Gittell and Weiss, 2004; Hitt *et al.*, 2007), hypothesising nested links of connection and constraint between an organisation’s environment, overall governance structure, sub-units, working practices, and (at all levels) groups and individual members. In this paper, we focus on the assumption that having different kinds of owners would lead organisations to:

1. Pursue substantively different goals.
2. Establish managerial regime which differ in:
  - The organisational structures which select and implement the organisation’s work processes and transmit the owners’ goals to them. Whether hierarchical or non-hierarchical, differently owned large organisations would, therefore, differ in the agency problems arising for the owners at the often-discussed interfaces between owners and managers (Bebchuk, 1999; Berle and Means, 1932; Demsetz and Lehn, 1985; Fama and Jensen, 1983), and between managers and production workers. Within a formal structure, network-like elements of “informal organisation” and market-like elements (an “internal market” in the strict sense) might also co-exist.
  - What aims and goals they prioritise, i.e. which command the most management attention and which the internal incentives (salaries, bonus, equity, status, career advancement, etc.) target: the “tight” elements in “tight–loose” management (Peters *et al.*, 1982).
  - The combinations of technologies of power (persuasion, material incentives, coercion, the “gaze”, technologies of the self, etc., Foucault, 1980) by which the owners, or managers as their proxy, control what the other members of the organisation do.
  - Organisational culture, including professional sub-cultures, i.e. their normative requirements for work practices and individual behaviour, justifications of the organisation’s work, its ownership and managerial practices, all expressed both verbally and in artefacts.

These assumptions imply that what differentiates differently owned organisations is above all the type of content of their goals, focus, exercise of power and legitimations; i.e., whether this content concerns above all finance, policies, occupational interests, etc., as the case might be.

To begin with goals, many studies assume *a priori*, and others report empirically, that “private” organisations’ dominant (not their only) objectives are financial, hence univocal and stable, whilst public organisations pursue more politically complex, mixed, unstable, vague, ambiguous, often predominantly symbolic objectives (Swiss, 2005). This difference has been conceptualised as a contrast of internal managerial incentives. “Private organisation” managers face, it is said, incentives to maximise “efficiency”, “social welfare” or “performance”, often equated with “financial performance”, “shareholder wealth” (Cragg and Dyck, 2003), output maximisation or profit seeking, hence with maximising income and/or minimising costs to the provider. Another conceptualisation imputes different “institutional logics” (each with their own regulative, normative and cultural-cognitive “pillars” (Scott, 2008)) to markets, corporations, the professions, the family, religions and the state (Greenwood *et al.*, 2010), or distinguishes a commercial from a public service ethos (Kirkpatrick *et al.*, 2017; Rainey and Bozeman, 2000). A third contrasts the corresponding organisational cultures. A comparison of public and private managers in Canada and Japan found that the longer managers had been in post, the more divergent their “values” became (Becker and Connor, 2005). Boyne’s (2002) narrative review of empirical studies of 13 hypothesised differences found that of the 8 differences about which secondary evidence then existed, the evidence supported 3: public organisations were more bureaucratic, public managers were less materialistic (had a stronger public service ethos) and had weaker organisational commitment than private sector managers (partly because many of them came from non-managerial professions).

Some studies (Alchian and Demsetz, 1973; Boyne, 2002; Kirkpatrick *et al.*, 2017; Meier and O’Toole, 2008) attribute differences in internal managerial regimes to differently owned organisations’ different operating environments, although further studies qualify these findings in various ways (Bozeman *et al.*, 1992; Rainey and Bozeman, 2000) or report common features of managerial regimes irrespective of ownership (Knott, 1993). However, one intention, and to a certain extent effect, of marketisation “reforms” has been to give all the differently owned providers in a particular quasi-market a similar organisational environment. Insofar as such “reforms” succeeded, one effect would be to constrain public organisations to mimic corporate structures and managerial regimes even without repositioning the public–private boundary.

Environment apart, boundary repositioning may also stimulate internal changes in managerial regime in consequence of ownership changes. Existing studies give a mixed picture of these endogenous differences. Some narrative reviews (Kirkpatrick *et al.*, 2017; Knott, 1993; Rainey and Bozeman, 2000) doubt whether certain formal differences between public and non-public management are large. Euske (2003) argues that all organisations need essentially the same accounting, costing, IT and “general’ functions’ systems irrespective of what those systems are used for. Rainey and Bozeman (2000) suggest that simplistic contrasts between private organisations’ univocal and public organisations” complex, “political” objectives are at best exaggerated. Public and private organisations managers have essentially the same capacity to reward employees (Simon, 1991). More generally, it has been argued (Berle and Means, 1932; Burnham, 1941) that managers are a separate social milieu, even class, with distinct economic interests of their own.

Nevertheless, certain differences in “private” and public organisations’ managerial regimes are also reported. By its “managerial regime”, we mean the ensemble of methods by which those who ultimately control an organisation do so (through what structures, strategies, incentives, managerial practices, ideologies and cultures), and in pursuit of what substantive goals. “Private” organisations’ greater managerial discretion and/or resources allow them:

1. To have proportionately more managers, especially finance managers (Aidemark and Lindkvist, 2004). Conversely, the “public firm” structures and management practices developed in NHS trusts increased the proportion of “strategic” managers but not that of specialist managers. Indeed NS trusts had a smaller (and decreasing) proportion of managers compared with the whole UK workforce (Kirkpatrick *et al.*, 2017) (a pattern possibly over reported by reclassifying clinician managers so as to claim reduced NHS “bureaucracy” (Hyde and Exworthy, 2016)).
2. Less formalised, standardised, “bureaucratic” managerial procedures (“red tape”), hence quicker completion of managerial tasks (Bozeman *et al.*, 1992), and less risk averse, more entrepreneurial (inventive, innovative, flexible and risk taking) decisions.
3. To mount stronger managerial challenges to restrictive practices and other “bureaupathologies” (Thompson, 1977), and face less pressure to take a short-term perspective in decision making.

Also, evidence that compatability with existing “values” (Greenhalgh *et al.*, 2004; Rogers, 1983) influences organisations’ selection and adoption of innovations implies different innovation patterns in organisations under different types of ownership. Hence, innovation, including organisational innovation, serves different purposes in private organisations (e.g. to promote competitive advantage, Hartley, 2005) than in public ones.

On the above assumptions, the content of managerial decisions would differ qualitatively between differently owned healthcare organisations, resulting in correspondingly different products, work processes (hence, user experience), technology adoption, workforces, information systems, internal incentives (both formal and informal) and managerial regimes.

## Research questions

### Aims

The many correlational studies of organisational differences on either side of the public–private boundary cannot, and often were not intended to, explore any such links between ownership and managerial regime. We lack reports of how management decisions, constraints and actions differ substantively – or not – between healthcare organisations on either side of the public–private boundary. The present study aimed to contribute to filling that gap, in doing so contributing to testing and as necessary revising a contribution towards an ownership theory of organisation.

### Research questions/hypotheses

Here we report some findings from a wider study (Sheaff *et al.*, 2016, p. 20) addressing the questions:

RQ1. How do the internal managerial regimes of differently owned healthcare providers differ, or not?

RQ2. In what respects did any such differences arise from differences in ownership or for other reasons?

Given the data available to us, we address the question in regard to the English NHS which, as explained above, now includes providers of quite diverse ownership.

## Methods

In the circumstances of the study, an observational systematic comparison of differently owned providers was the strongest feasible research design. The framework of comparison was the ownership theory outlined above. The English NHS is well suited to such a comparison because certain care groups are cared for by providers of contrasting ownership, but under the same payment systems. Those conditions control for gross differences in external payment and in technical task when comparing differently owned organisations (but not necessarily for case mix, for differently owned providers might be expected to select different case mixes, which would then be a consequence rather than a confounder of different ownership). To exploit these conditions, we assembled three qualitative sub-samples of case-study sites. In the English NHS different types of provider ownership are concentrated in specific services and, therefore, care groups. We, therefore, studied: CHS; out-of-hours primary care (OOH); and hospital planned orthopaedics and ophthalmology. Between them these services covered the “pure” (i.e. non-hybrid) types of ownership shown in Table I. We studied one instance of a provider of each type check marked in Table I.

Each column represents one sub-sample across which we could make a systematic comparison. Because all these services have high proportions of older patients with complex or multiple health conditions, we made that our focal care group. In each study site, we identified a key informant from whom we snowballed to other informants selected for relevance to that care group and to the conceptual framework outlined above. They typically included hospital directors or chief executives, medical directors, operations managers, finance managers, quality and performance managers, service managers and business development or marketing managers together with clinical leads in our tracer services, lead nurses or matrons and contract managers. Where available, we preferred informants who had worked in, and could, therefore, report the differences between, healthcare providers under different ownership. At the time of our fieldwork none of the corporate providers were above 10 years old (although the corporations owning them were older), so most of their staff had recently worked for the NHS. The social enterprises were equally new. The cooperatives, professional partnerships and voluntary hospital were older, but some informants in them had also worked in differently owned healthcare providers.

During 2015–2017 we assembled the data summarised in Table II. We used an omnibus semi-structured interview schedule, selecting for each interview the items that we particularly wanted to probe in light of what data we already had, and any ambiguities or contradictions in our data so far. We included informants’ accounts of other organisations provided that the informant was speaking from first-hand working experience. Taking the key informants’ advice as to which managerial documents were seminal to our research questions, we obtained those documents and content-analysed them.

The meetings included contract meetings (between provider and payer), clinical quality review meetings and combined quality and performance meetings (sometimes more than one per provider). With the interviewees’ consent, we audio recorded and transcribed the interviews. We pseudonymised the data throughout. Observations were recorded in contemporaneous field notes. Two researchers independently read the transcripts and field notes. A common coding framework was then applied to all data. Using a “framework” approach (Gale *et al.*, 2013; Ritchie and Spencer, 1994), we identified commonalities and differences in our data so as to explore inter-connections between different levels, occupations and interests. We drew descriptive inferences based on *a priori* and emergent themes before elaborating conceptual perspectives. The empirical themes explored informants’ descriptions of the effects of changes in provider ownership and of how others reacted to it, and their interpretation of its significance. Under each relevant main heading of our analytic framework, we present these themes below.

Each “case” (unit of analysis) was one type of provider (by ownership) for a given service (CHS, OOH or hospital speciality). For data integrity, we collated and triangulated all the data for each case from the sources listed in Table II. We used these case studies to populate a framework analysis whose categories reflected the ownership theory outlined above. This method was conceptually equivalent to a tabulation wherein each row was an ownership type and each column a category from the analytic framework. Data which did not fit into the framework were coded and separately analysed inductively to expose relevant themes which the initial framework omitted. Finally, we compared the resulting patterns against the analytic framework outline above, so as to expose any false or redundant assumptions: a falsificationist approach (Bitektine, 2008).

An NHS Ethics Committee approved the study (Reference No. 10/H0206/71) subject to informant anonymity.

## Findings

Following the above schema, we trace the main connections between types of ownership and the corresponding differences – and similarities – in managerial regime. The differences were mainly at the top (“governance”) levels of organisational structures; the similarities mainly at the lower (“work coordination”) levels.

### Goals and governance structures

Our study organisations’ governance structures reflected their diverse ownership. A Pan-European private equity group owned one for-profit corporation, an offshore holding company owned another, an American finance company the third. The NHS trusts were state owned. One of the CHS providers was a social enterprise in the peculiar NHS sense noted above. In contrast, the OOH provider which also called itself a “social enterprise” was essentially a cooperative in ownership, structure and internal management (and therefore categorised as such in this paper). The professional partnership was a partnership of (i.e. owned by) a group of general practitioners (GP). The not-for-profit hospital chain was collectively owned by its membership. Their Annual General Meetings elected the board, and the board decided who became members. In this circular way, its owners were self-selected.

#### Convergent aims, divergent objectives

All the case-study organisations irrespective of ownership had rather similar sets of broad, somewhat rhetorical aims about benefiting their patients and staff. Additionally, they had more specific objectives, stated by managers themselves, in managerial documents (e.g. annual reports), on websites and in other published materials. Where the case-study organisations differed by type of ownership was in what objectives they had, the balance of emphasis between objectives and the patterns of “tight–loose” control (Peters *et al.*, 1982), i.e. which aspects of managerial work were closely monitored, heavily incentivised and preoccupied managers in practice, and what was left to local discretion.

Both the for-profit corporation and the non-for-profit providers had clear, and prioritised, financial targets. The for-profit corporation hospital directors had a monthly business review by telephone with their line manager, covering quality, patient outcomes, patient experience, audits, scores against national norms “And of course the numbers comes into it as well” (Hospital Director CS2/M18). But as long as financial targets were met there:

[…] was quite a lot of autonomy on sites to make investment.

(operations manager, CS1/M07)

The not-for-profit hospital chain also had explicit financial targets, but to finance service developments not targets set by a holding company. The objectives of the OOH social enterprise similarly included making enough financial surplus to grow its “core business”. The voluntary hospital’s strategic objectives more vaguely stipulated a “reasonable” profit. Ex-NHS informants described both corporate and not-for-profit hospital budget management as being more rigorous than in the NHS.

The NHS trusts’ main objectives were also financial, but in the opposite way: financial retraction not growth. Our case-study NHS hospital had a £33m “Cost Improvement Programme” as its main objective. With a falling income and rising workload, the OOH trust planned to evolve into:

[…] a sort of network of arm’s length business units that sit within a wider structure […] our organisation won’t exist, and our board is absolutely clear [name] will not exist in a two to three year timescale because there will be a new entity that delivers an integrated primary care offer.

(CS15/M25)

Self-dismemberment was quite the opposite to the non-NHS organisations’ objectives.

The other social enterprises’ objectives tended to focussed more on non-financial matters. The CHS example formulated its objectives through workshops of 40 or 50 people that took up issues that emerged locally, which our informants thought made the social enterprise “ethical”. At the OOH cooperative:

[O]ur only concern is that patients are getting the best possible treatment, and if we can keep the cost to our GP members to a minimum, then all well and good.

(CS20/M55)

In the last year of our fieldwork, it returned its surplus to its member practices. Rather than having formal objectives, the GP partnership said:

[w]e have more of an ethos about how we want to practise medicine, and we would want partners to join us who share that ethos and […] continue doing what we do.

(CS31/M55)

Although they had no financial incentive to do so, they had developed an MSK pathway because:

[GP] and I looked at it, we know we can do so much better.

(CS31/M55)

In this case, a professional institutional logic prevailed over market logic.

#### Divergent governance structures

Top-level governance structures diverged by organisational ownership and objectives among the case-study organisations. As noted, the corporate examples all had a “financialised” (Montgomerie and Williams, 2009) structure designed to transfer payments from an operating company into a separate holding (owning) company. Our questions elicited that there was no direct interaction between the managers at the provider (hospital, clinic, call centre, etc.) level and the owning companies, which in all cases seemed rather distant from the service provider managers’ day-to-day practical coordination activity.

Strong “quasi-hierarchical” (Exworthy *et al.*, 1999; Sheaff and Schofield, 2016) accountability chains (Day and Klein, 1987) linked our case-study NHS trusts to NHS and local authority payers, NHS England, the Department of Health and ultimately Parliament. NHS chief executives’ careers and continuity in post depended above all upon achieving the targets (including cost control) most important to government, the so-called (Bevan, 2009) “targets and terror” regime: a politicised structure. Informants at one SE described how in their NHS days they were:

[…] required to report every nut and bolt back through an accounting chain and back through the performance management chain.

(CS23/M37)

Having no external governance structure, the voluntary hospital, not-for-profit chain and CHS e-social enterprise could pursue whatever “mission” their members chose: a mission-based structure. Doctor-owned organisations had correspondingly doctor-controlled structures. Two-thirds of the OOH cooperative’s Management Council was elected by the GP members, who included every GP working for this provider, and appointed a medical director, chief nurse, chair, two non-executive directors and various sub-committees. The general practice providing CHS was jointly controlled by its GPs, one of whom (the “managing partner”) managed the employed non-medical staff.

### Convergent coordination structures

#### Convergence on hierarchy

Doctor-controlled organisations half-excepted, all the case-study organisations had a hierarchical, bureaucratic structure for coordinating everyday service production, hence largely similar daily managerial work for that. Former NHS managers said their day-to-day work had changed little with the change in ownership. Marketing apart, the range of management functions (HR, finance, IT, etc.) and “line” management (of operations, nurse, therapy, administrative and ancillary services managers) was similar. Although ex-NHS informants often described some NHS trust decision making as slower, labyrinthine, frustrating and more cumbersome, it nevertheless did not always achieve tighter control:

[…] in an NHS organisation you just carry on overspending or without there being proper demand and controls in place.

(CS23/M34)

Most of our ex-NHS informants described themselves, and their subordinates, as being more accountable than before in organisations under corporate, not-for-profit and voluntary ownership. They meant internally accountable to their own managers. However, Checkland *et al.* (2013) also found that the emergence of clinical commissioning groups (GP-controlled bodies who selected and paid most NHS-funded providers) increased the complexity – rather than the clarity – of the external, inter-organisational accountability chains in which NHS-funded providers found themselves, both “upwards” (quasi-hierarchically) and horizontally to collaborators which would include other statutory bodies and commercial partners.

Because it requires fewer tiers of management, a smaller organisation can have a “flatter” structure, shorter chains of command and simpler management arrangements than a large organisation with just the same spans of control. Interviewees described how the corporate providers’ smaller size allowed:

[…] the medical and nursing staff, and everyone else for that matter, but for the clinicians to actually be much more involved in the running and the change management of a smaller organization.

(CS2/M19)

Corporations, non-profit and voluntary organisations’ hierarchical coordination structures accommodated doctors in three different ways. NHS-employed consultants most often had admitting rights, but one corporate hospital also directly employed its orthopaedic surgeons and sub-contracted its ophthalmologists from a medical “chamber”.

#### The semi-democratic exception

The controlling bodies of the social enterprise OOH provider, cooperative and partnership CHS democratically represented the doctors in them. In effect, they collectively coordinated their own work by volunteering for shifts and finding other doctors to fill any gaps. All three organisations coordinated the non-doctors’ (receptionists, drivers, administrators, managers, nurses, mental health workers, counsellors and psychologists) work by employing them in a hierarchy managed by a chief executive (or equivalent), hence ultimately the member doctors. Our informants saw this structure as able to make quick decisions, “able to evolve and adapt quickly” (CS20/M55), and with low management costs (below £150,000 annually for the GP cooperative).

## Discussion

This study was empirically limited to just one sector in one country, although within that sector our case-study organisations were typical of their kinds. Other studies corroborate that financialised structures are common among UK healthcare corporations, especially the largest, and the seven largest take 88 per cent of the NHS-funded inpatient work which is not done by NHS trusts (Appleby, 2015). Governance structures for NHS trusts and social enterprises are nationally prescribed. Professional partnerships are by far the commonest form of general practice organisation (Sheaff, 2013). The ownership and structure of the OOH cooperative described here was also similar to others (Hallam and Henthorne, 1999; Sheaff *et al.*, 2012), as were the not-for-profit and voluntary hospitals we studied. Nevertheless, we studied only one professional partnership organisation so must be cautious in generalising from that case. An analysis of the study sites’ catchment populations and case mix (obtainable from the authors) showed that none of our hospital study sites were outliers in those respects. (Such data are not collected for OOH and CHS services.)

A second empirical limitation is that we have focussed on formal structures, omitting to varying extents other technologies of power within differently owned organisations: informal organisation; ideology (including culture, organisational identity and disciplinary control); technologies of the self; the interactions of multiple cultures and institutional logics (Reay and Hinings, 2009); and resistance to managerial and owners’ control. We have focussed on “downward” influence rather than any reciprocal “upwards” (e.g. technological) connections between organisational ownership and managerial regime (for instance, whether cooperatives or partnerships are more resistant to becoming corporations than public organisations are), or influences across formal organisational boundaries.

The paper does not consider how far these ownership differences affected different organisations’ work processes, outputs and interactions with their environments, and with what consequences. These topics merit further comparison across differently owned organisation. In these ways, the present study points towards a wide ranging further research. Participants’ narratives of organisations’ formation and development – a historical approach – would be a strong research design for testing and revising an ownership theory of organisation. Testing a multi-level theory would also appear to require a multi-level research design (Hitt *et al.*, 2007).

Nevertheless, our findings tend to support the idea that even insofar as managerial structures of differently owned organisations have formally similar structures (managerial “functions” or specialisms, uniprofessional sub-hierarchies, project teams, etc.), the substantive uses to which they were put differ in important respects. Such differences might explain why superficially similar managerial actions may have different impacts in differently owned organisations. For instance, publicly owned schools in Denmark adopted “performance” management earlier and more widely, but to less effect in terms of pupils’ educational grading, than private not-for-profit schools (Hvidman and Andersen, 2014). Such findings give further, indirect corroboration. Contrary to our initial expectations, and perhaps those of the cruder theories about the connections between ownership (on the one hand) and organisation structures and managerial regimes (on the other), these connections differed at different organisational levels.

A managerial implication is that these findings recommend caution in copying organisational structures and managerial practices from one organisation to others under different kinds of ownership. In our study, sites external promotional activities, for example, served different purposes in the corporations than the NHS trusts (to increase and to reduce demand, respectively). As another example, HR management worked to casualise and de-skill work in the corporations but to maintain (doctors’) job security and centrality in the professional partnership and cooperatives. A theoretical implication is that organisations’ internal managerial workings can only be understood by reference to their owners’ aims. Research which abstracts from those aims ignores the central motivation, rationale and aims of managerial work in the organisations being studied. Theoretical questions also arise about the connections between ownership and work processes: which constrains (some would say: “determines”) which? And with what consequences, for the activities and outcomes of interest to health policy?

## Conclusions

So far as we are aware this study is one of very few to compare systematically the internal managerial consequences of diverse ownership of healthcare providers. On balance, our data support the view that the management of differently owned organisations (e.g. those on different sides of the public–private boundary) remains “fundamentally alike in all unimportant respects” (Sayre, 1958), provided we take hierarchical coordination structures and their associated managerial functions (accounting, IT, etc.) as the unimportant respects. Even so, “alike” would be an overstatement because hierarchical coordination was not ubiquitous in the doctor-owned organisations. Our data suggest that whilst ownership does make a difference to coordination structures, the larger difference is not the one between organisations on either side of the private–public ownership boundary. Rather, the boundary that makes a difference to it is the boundary between organisations owned by at least some of those who directly produce the organisations’ outputs (goods or services), and organisations which are not. In the former case, their (in this case, doctors’) work was controlled and coordinated in a more-or-less democratic, self-governing ways. Conversely, the “important respects” would be taken as the differently owned organisations’ governance structures, managerial regimes and their operationalised, most tightly managed objectives (those upon which internal managerial incentives are brought to bear, rather than their broad and rhetorical aims).

Together these conclusions offer some *prima facie* evidential support which would contribute to grounding an ownership theory of organisation (not yet fully developed). Type of ownership does appear, overall, to make a difference to at least some important aspects of an organisation’s governance structures and managerial regime. For the broader field of health organisational research, these findings highlight the importance of the owners’ agency in explaining organisational change. Healthcare organisational research which sufficiently recognised that point would involve studying, first, power within organisations, i.e. the ways in which the default assumption that the owners are the most powerful actors within a given organisation have to be understood, nuanced or modified, case by empirical case. It would attend to the ways in which managerial legitimations of organisational change simultaneously express the owners’ interests and formulate them so as to persuade non-owners to accept and pursue them. Also it would attend to how the legitimations of externally proposed system interventions (e.g. policy demands for greater care “integration”) relate to the relevant organisations’ owners’ interests and wishes, and whether, therefore, these legitimations motivate these owners to implement, ignore or resist the proposed changes. In these ways, organisational ownership bears upon the governance and public accountability of health systems as a whole. Our findings also call into question the practice of copying managerial techniques (and “fads”) across the public–private boundary, and the idea that repositioning that boundary is purely a “back-end” change, without consequence for health policy outcomes.

## Figures and Tables

**Table I:** Qualitative sample: maximum variety by ownership type

	Hospital orthopaedics and ophthalmology	CHS	OOH
Corporate for-profit	✓	✓	✓
Proprietary			✓
Public	✓	✓	✓
Cooperative			✓
“Social enterprise”		✓	✓
Other Not-for-profit	✓		
Professional partnership		✓	

**Table II:** Data collected

	Interviews	Other material
Organisational case studies	83	212 documents, 226 media reports
Commissioning meetings		15 observations (27 h)
Patients	89	5 focus groups (38 participants), 5 individual patients/representatives
Contextualisation data	n/a	Extracts from Hospital Episode Statistics, Office of Population Census and Surveys
Secondary data	n/a	283 published papers, 34 research reports
